# Technology-facilitated gender-based violence against women and girls in sub-Saharan Africa: A scoping review

**DOI:** 10.1371/journal.pone.0354526

**Published:** 2026-08-19

**Authors:** Yonas Abebe Tarefa, Abdul-Fatawu Abdulai

**Affiliations:** 1 School of Nursing and Midwifery, Institute of Health Science, Wollega University, Nekemte, Ethiopia; 2 School of Nursing, University of British Columbia, Vancouver, British Columbia, Canada; Washington University in St. Louis, UNITED STATES OF AMERICA

## Abstract

**Background:**

Technology-facilitated gender-based violence is extension of existing power dynamics and patriarchal systems that continue to oppress and marginalize women and girls. However, the mechanisms through which technology enables such violence and its effects on victims remain underexplored, particularly in the context of sub-Saharan Africa.

**Objectives:**

This review aimed to examine how technology facilitates gender-based violence and to explore its impact on women and girls in sub-Saharan Africa.

**Methods:**

This scoping review was conducted following the Joanna Briggs Institute (JBI) methodological guidance for scoping reviews and adhered to the PRISMA-ScR checklist for standardized reporting. The literature search was conducted across multiple databases, including EBSCO, Scopus, PubMed, ScienceDirect, other source including Google Scholar, and AJOL. Eligibility criteria were defined using the Population, Concept, and Context (PCC) framework. Thematic analysis was employed to identify and organize emerging concepts and themes. Data were analyzed using Atlas.ti version 7.5.4 and the results were presented in tables, narrative descriptions, and diagrams.

**Result:**

From a total of 344 articles identified through databases and other sources, 17 studies met the inclusion criteria and were included in the review. The review finding shows how technologies enable gender-based violence. These include the anonymity of perpetrators and the increased accessibility of victims, coercive control through digital surveillance, the viral spread of harmful content leading to public shaming, and specific features of digital platforms that facilitate abuse. Another finding was effect of technology facilitates gender-based violence (TFGBV)on victims. Reported effect include psychological harm, negative effects on academic and educational performance., social and reputational damage, as well as barriers to seeking help and long-term trajectories like depression and poor academic performance

**Conclusion:**

These findings indicate that technology plays a substantial role in facilitating gender-based violence, and the impact on victims is significant.

**Protocol Registration:**

OSF (https://doi.org/10.17605/OSF.IO/47ZB8).

## Introduction

In an increasingly digitalized world, technology is transforming not only modes of communication but also the very contexts of social interaction. For women and girls, this transformation presents both opportunities and risks. While digital platforms can amplify voices, strengthen networks, and expand access to support, they are also increasingly weaponized as tools of harassment, surveillance, and abuse [1,2]. Technology-facilitated gender-based violence (TFGBV) refers to acts of violence that are committed, assisted, aggravated, or amplified either partially or entirely through the use of information and communication technologies (ICTs) [3]. This includes harassment, exploitation, bullying, surveillance, and the non-consensual distribution of content, carried out via social media platforms, messaging services, email, and other digital spaces reflecting the extension of offline gender inequalities into digital environments [4,5].

Unlike traditional gender-based violence, which has been rooted in physical spaces and often constrained by locality and visibility, TFGBV transcends borders and anonymity. Digital technologies allow perpetrators to act with concealment, reach wider audiences, and sustain abuse across time and space [6–9]. Online harms are further magnified by the persistence and permanence of digital content, which can be rapidly shared, archived, and re-circulated, leaving victims exposed indefinitely [8,10]. This erodes the boundaries of safety, making abuse possible even in private spaces such as victims’ homes [9,11].

Globally, TFGBV is widespread and escalating. Estimates suggest that nearly one in three women may experience technology-facilitated sexual violence, though prevalence varies by region [12]. In Canada survey of undergraduate student shows, 84.3% of students reported experiencing some form of TFGBV [13]. UN Women report found that 60% of women in the Arab States and more than half of women in Eastern Europe and Central Asia have faced such violence. In Sub-Saharan Africa, 28% of women report experiencing online abuse [14]. These figures, however, are likely underestimates given the prevalent underreporting and limited regional data.

Despite its growing significance, research on TFGBV in developing contexts remains limited. Legal frameworks lag behind technological realities, institutional and social barriers discourage reporting, and the cross-border nature of online abuse complicates prevention [11,15–20]. In sub-Saharan Africa, where gender inequality and digital divides are common, the absence of robust evidence risks interventions that are misdirected, ineffective, or unsustainable. Therefore, this scoping review intends to map evidence and explore how technologies facilitate gender-based violence and explores its effects on victims. It is the first to systematically address this issue in Sub-Saharan Africa. By filling a critical knowledge gap, the review provides essential evidence to guide effective interventions and policies, while advocating for digital safety, gender equity, and advancement of human rights across the region.

## Review questions

1. How does technology facilitate gender-based violence against women and girls in sub-Saharan Africa?
2. What are the effects of technology-facilitated gender-based violence against women and girls on victims in sub-Saharan Africa?

## Methods

We adopted a scoping review methodology to synthesize the evidence on technology-facilitated gender-based violence. Technology-facilitated gender-based violence is a relatively emerging area of research in sub-Saharan Africa. Therefore, we preferred to conduct a scoping review to investigate the scope of the literature, clarify the concept, and investigate the research conducted [21]. This review adheres to Joanna Briggs Institute (JBI) methodological guidance of conducting scoping review [22]. To enhance the reporting standard of the scoping review, the Preferred Reporting Items for Systematic Reviews and Meta-Analyses Extension for Scoping Reviews (PRISMA-ScR) were followed [23](see S1 File). This scoping review adhered to a pre-defined protocol registered on the Open Science Framework (OSF) prior to the commencement of the review process (https://doi.org/10.17605/OSF.IO/47ZB8).

### Search strategy

Following JBI recommendations, our search was conducted in three phases [22]. First, an exploratory search was carried out in EBSCOhost and Scopus using the keywords *‘technology’* AND *‘gender-based violence’*. The purpose of this initial search was to identify relevant text words, synonyms, and index terms to inform the development of a comprehensive search strategy. Second, the refined search strategy combining text words and controlled vocabulary terms, including general and specific descriptors was applied across all selected databases (EBSCOhost, Scopus, PubMed, and ScienceDirect). For example, the following search string was used in PubMed: (“Gender-Based Violence”[Mesh] OR “Sexual Trauma”[Majr] OR bullying OR harassment OR “online abuse”) AND (“Smartphone”[Mesh] OR “Mobile Applications”[Mesh] OR “Information Technology”[Majr] OR “Internet Use”[Majr]) AND “Africa South of the Sahara”[Mesh]. Third, to ensure completeness, supplementary searches were conducted in additional sources, including Google Scholar, and African Journals Online (AJOL). No restrictions were applied regarding publication year, language, or age of participants. Detailed search strategies for each database are provided in S2 File. The same search strings were also adapted and applied using the names of all Sub-Saharan African countries as listed by the World Bank [24]. The database search was started on September 01,2025 and completed on November 15, 2025.

### Eligibility criteria

In accordance with the JBI guidelines for scoping reviews, the eligibility criteria for included studies were framed using the Population, Concept, and Context (PCC) framework.

#### Population.

This review focuses on women and girls residing in sub-Saharan Africa, examining their experiences with TFGBV. There are no restrictions based on age, disability, marital status, occupation, socio-economic status and sociodemographic information. To be included, studies must explicitly disaggregate data by gender and identify GBV acts facilitated through digital technologies, where the violence is motivated by the victim’s gender and intended to harm or exploit women and girls. Studies that focus solely on men, or that are gender-neutral/gender-blind, are excluded, as the review is specifically concerned with the gendered nature of violence targeting women and girls.

#### Concept.

This review explores technology-facilitated gender-based violence (TFGBV) against women and girls, a form of gender-based violence in which digital technologies and online platforms are used to enable, facilitate, amplify, or perpetrate violence targeting individuals because of their gender. The review specifically focuses on violence directed toward women and girls through widely accessible digital technologies such as smartphones, tablets, computers, social media platforms, messaging applications, and online communication tools, including but not limited to Facebook, Instagram, TikTok, WhatsApp, Telegram, X (formerly Twitter), and dating applications. Studies were excluded if they addressed gender-based violence without a technological component or examined technology-assisted violence that was not gender-motivated or not specifically directed toward women and girls.

#### Context.

This review is limited to sub-Saharan African countries, as defined by the World Bank classification [24]. Studies from other African regions or non-African countries were excluded. For multi-country studies, only findings that are specifically reported for sub-Saharan African countries were considered. Studies where sub-Saharan African data could not be disaggregated were excluded.

## Study selection and data extraction

This review included primary studies such as observational studies, cross-sectional studies, case-control studies, and case study and case reports. Findings from qualitative, quantitative and mixed studies were included. Abstracts, non-research letters, editorials, and reports from humanitarian organizations were excluded.

Search results from all databases were exported into EndNote X8. Search results from Google scholar and AJol were added manually and duplication was removed. Then, all retrieved studies were screened based on their title, followed by abstract screening and then full text screening depending on inclusion and exclusion criteria outlined. All screening and review performed by two independent reviewers. Relevant data from included studies was extracted by two independent reviewers. Excell spreadsheet which prepared in line with JBI data extraction recommendation [25]used for relevant data extractions. The extracted data included specific details about study characteristics authors, study design, objectives, samples, year, country, and key findings relevant to the review questions. Throughout the data extraction process, the draft data extraction tool was changed and improved as needed. Reviewer disagreements were settled through discussion with a third person outside of the study team.

### Data analysis

The search and screening process was documented using the PRISMA-ScR flow diagram [23]. Data from the included studies were charted and synthesized to map the breadth, characteristics, and patterns of evidence related to technology-facilitated gender-based violence (TFGBV) against women and girls in sub-Saharan Africa.

In addition to summarizing study findings, the review mapped key study characteristics, including country of origin, study population, sample characteristics, methodological approach (qualitative, quantitative, or mixed methods), study design (e.g., cross-sectional, qualitative), and type of data source (primary or secondary data). This mapping approach enabled a clearer understanding of how evidence on TFGBV against women and girls has been generated across different contexts and populations within the region.

The findings are presented through narrative thematic synthesis. Each theme is supported by descriptive summaries and the number of studies contributing to that theme to enhance transparency and demonstrate the scope and distribution of the evidence base. Qualitative findings were analyzed thematically using inductive coding in Atlas.ti version 7.5.4, allowing concepts and patterns to emerge directly from the data without reliance on pre-established frameworks. Quantitative findings were synthesized narratively by summarizing prevalence estimates, methodological patterns, study designs, and major statistical trends across studies.

Where appropriate, findings from qualitative and quantitative studies were integrated using a data-based convergent synthesis approach, with evidence synthesized thematically according to the review objectives. This integrated approach facilitated a comprehensive mapping of the manifestations, consequences, and methodological characteristics of TFGBV against women and girls in sub-Saharan Africa and is consistent with JBI methodological guidance for mixed-methods reviews [26].

## Result

### Screening and selection of included studies

**Fig 1:**
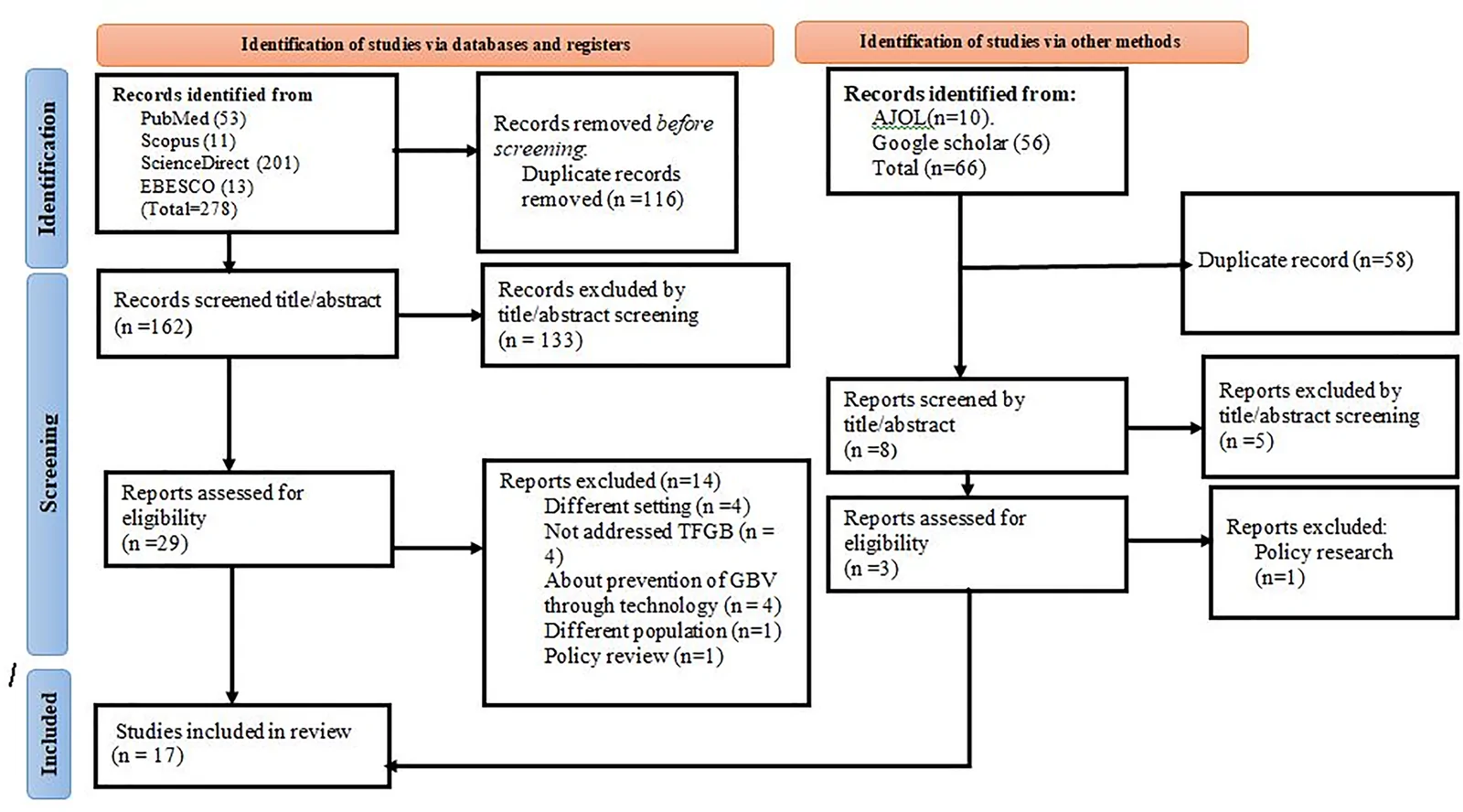
PRISMA flow diagram of included studies.

The study selection process is presented in the PRISMA flow diagram (Fig 1). A total of 278 records were identified from PubMed (n = 53), Scopus (n = 11), Science Direct (n = 201), and EBSCO (n = 13). An additional 66 records were identified through AJOL (n = 10) and Google Scholar (n = 56), resulting in a total of 344 records. After removing duplicates (n = 174; 116 from the main databases and 58 from AJOL/Google Scholar), 170 unique records remained for screening, comprising 162 from the main databases and 8 from AJOL/Google Scholar.

From the main databases, 162 records were screened by title and abstract, of which 133 were excluded. The remaining 29 full-text articles were assessed for eligibility. Fourteen were subsequently excluded for the following reasons: conducted in settings outside sub-Saharan Africa (n = 4), did not address TFGBV specifically (n = 4), focused on technology-based prevention of GBV rather than TFGBV (n = 4), Targeted a different population (social media posts without differentiating gender) (n = 1), and policy review (n = 1) (see S3 File).

From the AJOL/Google Scholar set, 8 unique records were screened by title and abstract, of which 5 were excluded. Three full-text articles were assessed for eligibility, and 1 was excluded as a policy review, leaving 2 eligible studies.

In total, 17 studies were included in the scoping review.

### Characteristics of included studies

This review includes 17 study reports, from 14 are original research. All these studies, published between 2019 and 2024, show that the topic is an emerging issue. Seven studies were conducted in Kenya [27–32]. Two studies were from Uganda [33,34], and two were from Zimbabwe [35,36]. The remaining studies were conducted in Tanzania, Nigeria, Malawi, other sub-Saharan African countries, and multi-country studies including Rwanda, South Africa, and Uganda, as well as one study from Ghana.

In terms of method, approximately eight studies employed mixed methods, four used qualitative approaches, and the rest employed quantitative design. Most of the studies were conducted among university students**.** Detail characteristics of included studies were documented in (Table 1). The included literature examined various categories of technology-facilitated gender-based violence, documented cases including cyberbullying, cyber harassment, and cyberstalking and others (see Table 2).

**Table 1:** Characteristics of included studies.

Authors/year	Country	Objectives	Design	Data collection methods	Summary of findings
(Kavishe, 2024) [37]	Tanzania	This study aimed to examine female students’ awareness of gender-based violence and online violence, and to demonstrate how digital tools reinforce the negative effects of gender-based violence among this population	Mixed	• Survey • FGD • Key informant interview	• Awareness: Aware of gender-based violence, but limited understanding of root causes of online GBV. • Role of Technology: ◦ Conceals perpetrators ◦ Increases victim accessibility ◦ Multiplies of violent incidents ◦ Extends aggression in space and time ◦ Provides a platform for female students’ self-objectification.
(Brown et al., 2024) [33]	Uganda	To explore the intricate nature of TFGBV in Uganda, investigate the effectiveness of Uganda’s existing legal and policy framework on TFGBV, examine the impact on victims, and propose comprehensive strategies for its effective regulation	Mixed	• Survey • Desk Research • In-depth interview	• Widespread Technology-Facilitated Gender-Based Violence (TFGBV) • Root Causes: Systemic legal, cultural, and institutional gaps • Recommendations: Coordinated, gender-sensitive, multi-stakeholder approach needed.
(WOUGNET et al., 2019) [34]	Uganda	To assess tech-related VAW, their implications and solutions proposed.	Mixed	• FGD • Key informant Interview • Document and records • Video	• Low Awareness of Reporting Mechanisms: Significant lack of knowledge regarding reporting avenues for tech-assisted VAW among respondents. • Concept Familiarity: Respondents generally familiar with the concept of tech-assisted VAW.
(Mayoyo, Malenya, et al., 2020) [27]	Kenya	To explore Kenyan undergraduate university students‟ perception of the concept of Cyber Dating Abuse and its prevalence among them	Mixed	• Survey • FGD	• High Prevalence of Cyber Dating Abuse: The study indicates a high prevalence of Cyber Dating Abuse among respondents • Dominance of Coercive Control: Coercive control was identified as the most pervasive form of abuse within Cyber Dating Abuse. • Ambivalence Towards Cyber Dating Abuse: Respondents exhibited mixed feelings and uncertainty about what constitutes Cyber Dating Abuse. • Normalization of Abusive Acts: A majority of respondents perceived many acts of Cyber Dating Abuse as “normal” rather than abusive. • Contextual Perception of Abuse: Just over a third of respondents believed the abusiveness of certain acts depended on the context and the type of relationship. • Justification of Abuse: Misguided perceptions led to the justification of Cyber Dating Abuse, contributing to its prevalence.
(Ndawana & Chisambiro, 2024) [36]	Zimbabwe	To examines how digital technology, especially social media platforms, shaped the risk of gender-based violence (GBV) in Zimbabwe during the coronavirus (COVID-19) pandemic	Qualitative	• Documentary analysis • Key informant interviews	• Anonymity Empowers Perpetrators: Social media provides anonymity to online GBV perpetrators. • Propagation of GBV: social media promotes and propagates multiple cases of GBV. • Perpetuation of Gender Inequality: social media perpetuates gender inequality. • Unequal Access for Victims: social media is not accessible to all victims of GBV. • Strong & Complex Relationship: The relationship between social media and GBV is strong and complex.
(Mayoyo et al., 2023) [28]	Kenya	To explore the use of digital media by undergraduate students in selected universities in Nairobi City County in perpetrating different types of violence as outlined in Michael Johnson’s typology of intimate partner violence	Mixed	• Survey • FGD	• WhatsApp as a Key Platform: WhatsApp was the most frequently used platform for perpetrating intimate partner violence. • Comprehensive Abuse Forms: The violence perpetrated covered all four forms of abuse (physical, sexual, psychological/emotional, and financial). • Pervasiveness of Coercive Control: Coercive control violence, involving monitoring and control, was the most pervasive form of abuse. • Digital Technology Facilitation: The proliferation and accessibility of user-friendly digital technologies and social networks facilitate the perpetration of CDA.
(Akeusola, 2023) [38]	Nigeria	This study investigates the impact of TF-SV on well-being and academic performance of undergraduate students in Nigerian tertiary institutions	Quantitative	Survey	• Positive Correlation: TF-SV & Psychological Distress: Exposure to Technology-Facilitated Sexual Violence (TF-SV) is positively correlated with psychological distress (anxiety, depression). • Negative Correlation: TF-SV & Self-Esteem: Exposure to TF-SV is negatively correlated with self-esteem. • Negative Correlation: TF-SV & Academic Performance: Exposure to TF-SV is negatively correlated with academic performance (GPA).
(Malanga, 2021) [39]	Malawi	To investigate the prevalence of cyber violence against women in Karonga district of Malawi.	Quantitative	Survey	• Rising Cyber Violence in Malawi: Women in Malawi are experiencing increasing cyber violence. • Forms of Cyber Violence: Cyberbullying, harassment, and revenge pornography are common forms. • Platforms Used: Facebook and WhatsApp are key platforms for perpetrating cyber violence. • Motivations of Perpetrators: Revenge, jealousy, and political agendas drive perpetrators. • Consequences of Cyber Violence: Social, psychological, economic, and physical harm are inflicted on victims. • Underreporting: Lack of awareness and cultural factors contribute to underreporting of incidents. • Significant and Growing Problem: Cyber violence against women is a significant and growing problem in Malawi.
(Mathew et al., 2019) [29]	Kenya	This study examines the prevalence of cyberbullying behaviors among adolescents in sampled schools in Westland’s Sub-County, Nairobi County.	Quantitative	Survey	• Prevalence of Cyberbullying & Victimization: 14% of respondents participated in cyberbullying, while 23% experienced cyber-victimization. • No Correlation: Compulsive Internet Use & Cyberbullying/Victimization: No association was found between compulsive internet usage and either cyberbullying or cyber-victimization. • Positive Correlation: Cyberbullying & Cyber-Victimization: A significant positive correlation exists between cyberbullying perpetration and cyber-victimization, suggesting victims may become perpetrators. • Gender Impact: Gender, not age, significantly impacts the relationship between internet use and cyberbullying/victimization behaviors. • Noteworthy Prevalence: The study highlights a noteworthy prevalence of cyberbullying and cyber-victimization among teens in Westland Sub-County, Nairobi County.
(Mafa et al., 2020) [35]	Zimbabwe	To explore the dynamics surroundings revenge porn as a phenomenon thwarting the efforts of women-NGOs in their fight against gender inequality and women exclusion	Qualitative	• FGD • Key informants	• Revenge Porn Harms in Zimbabwe: Revenge porn causes severe psychological, social, and economic harm to women in Zimbabwe. • Driving Factors: Patriarchal attitudes and the misuse of social media fuel the problem. • Exacerbating Factors: Weak legal frameworks and societal stigma worsen the issue • Need for Action: Urgent need for legal reforms, public education, and psychosocial support to protect women’s rights.
(Simon & J., 2021) [30]	Kenya	This study aimed at analyzing how the use of smartphones interrelated with the exchange of sexually inclined messages in daily social life of middle level college (MLC) students in Nairobi, Kenya	*qualitative research design*	• FGD	• Reciprocal Influence on Sexting: Students’ sexting views and behaviors are influenced by, and also influence, their romantic partners’ sexting activities (both online and offline). • Negative Beliefs About Sexting: Many students hold incorrect and negative beliefs about the role of sexting in romantic relationships.
(Makinde et al., 2021) [40]	Sub-Saharan Africa	To assess the Nature of Technology-Facilitated Violence and Abuse among Young Adults in Sub-Saharan Africa	*Literature review and survey*	• Survey	• Variety of TFVA in SSA: Sub-Saharan Africa experiences various forms of technology-facilitated violence and abuse (TFVA), including cyberbullying, cyberstalking, and image-based abuse. • Both Genders Experience TFVA: Both young men and women experience TFVA. • Common Form: Unwanted Sexually Explicit Content: Unwanted sexually explicit content is the most common form of TFVA. • Gendered Differences in TFVA: Women report more unwanted sexual requests, while men report more violent threats. • Coping Mechanisms: Common coping strategies include blocking abusers and deleting profiles.
(Odhiambo Ogolla et al., 2022) [31]	Kenya	To investigate factors influencing the occurrence of cyberbullying on Facebook among undergraduate students in Kenyan Universities	*Mixed*	• Survey • Key informant • FGD	• Cyberbullying on Facebook Common Among Kenyan Students: Most undergraduate students in Kenyan universities have experienced cyberbullying on Facebook. • Influencing Factors: Number of Facebook friends, level of interaction on Facebook, and certain demographic attributes influence cyberbullying prevalence.
(Strategy Q3, 2024) [41]	Indonesia, Jordan, Lebanon, Morocco, Rwanda, South Africa, and Uganda	To Understand the nature of technology-facilitated gender-based violence (TFGBV).	• *Qualitative*	Key informant interviews	• Recognition of TFGBV: Under-recognized as a legitimate form of gender-based violence (even among lawmakers and law enforcement). • Interconnectedness: Online and offline violence are interconnected; digital abuse often escalates to physical harm. • Root Causes: Patriarchal norms and moral policing. • Legal Framework: Legislation exists but is fragmented, inconsistently enforced, and sometimes used against survivors. • Reporting: Weak mechanisms; survivors face stigma, legal risks, and institutional mistrust.
(Mayoyo, Ogeno, et al., 2020) [32]	Kenya	To explore kinds of communication technologies used in perpetrating different forms of Cyber Dating Abuse.	*Mixed*	• Survey • 6FGD	• Common Digital Media Usage: Respondents primarily used smartphones for calls, text messages, and instant messaging on social media. • WhatsApp Predominance: Instant messaging on WhatsApp was the most frequent platform used to perpetrate Cyber Dating Abuse. • Digital Interaction Facilitates Victimization: Social interaction via digital media enabled offenders to find and target suitable victims (partners). • Increased Visibility and Accessibility: Extended digital media use by victims increased their visibility and accessibility to offenders. • Positive Correlation with Online Time: A statistically significant positive correlation (r = 0.20, p ≤ 0.001) was found between time spent online and Cyber Dating Abuse victimization. • Likelihood of Persistence: Due to the evolving nature of digital media and continued youth usage, Cyber Dating Abuse victimization is likely to persist.
(Odhiambo Ogolla et al., 2022) [31]	Kenya	To investigate types of cyberbullying experienced by undergraduate students on Facebook in Kenyan universities.	*Mixed*	• Survey • Key informant • FGD	• TFGBV on Facebook Affects Kenyan Students: Kenyan university students on Facebook experience technology-facilitated gender-based violence (TFGBV). • Disproportionate Impact on Women: Female students are disproportionately affected by shaming (50.6%) and cyberstalking (43%). • Common Forms of Abuse: Identified forms include impersonation, blackmail, and appearance-based harassment. • Recommendations: Universities should raise digital safety awareness and offer support services to address these challenges.
(Sam et al., 2019) [42]	Ghana	To examine the prevalence of cyberbullying among high school and university students in Ghana, and its consequences	*Quantitative*	• survey	• High Prevalence of Technology-Assisted Cyberbullying in Ghana: Most Ghanaian youth experience cyberbullying via technology. • Modest Psychological Impact Suggests Normalization: Despite high rates, psychological consequences are modest, suggesting possible normalization of technology-assisted GBV. • Unexpected Factors May Contribute: Factors like openness and self-enhancement may increase vulnerability to technology-assisted GBV.

**Table 2:** Summary of types of TFGBV reported in literatures.

Types TFGBV	Description of the types of violence
***Cyber-harassment (online Hate Speech)***	Repeated, harmful, or threatening online messages intended to cause psychological or emotional harm. It is broadest of these two terms (Cyberstalking, cyberbullying fall under the umbrella of cyber harassment). Studies reporting [34,36,37,39,42]
***Cyber-bullying***	An aggressive and deliberate act conducted through electronic means by an individual or group, repeatedly and over a period of time, targeting a victim who is unable to easily protect themselves [43]. Studies reporting [29,31,42]
***Cyber-stalking (sometimes called digital controlling)***	It is a form of stalking carried out through technology, involves repeated unwanted messages, threats to reveal confidential information or harm the victim, and the use of technology to track the victim’s activities and location. Studies reporting [27–29,31,32,34,37,39,41,44,45]
***Non-consensual Intimate-Image Distribution (NCII); “Revenge Porn”***	Distribution of intimate images without consent, sometimes weaponized after initially consensual sharing. Studies reporting [30,32–34,36,39,40].
***Sextortion***	Coercion to share sexual content under threat, such as blackmail or exposure. Studies reporting [37,39–41].
***Cyber-dating Abuse (Digital Intimate-Partner Violence)***	Use of digital tools to monitor, control, or psychologically harm a romantic partner. Studies reporting [27,28,32,39,40]
***Defamation, Doxxing and Public Shaming***	These are interrelated concept involves the dissemination of false information, or non-consensual disclosure of personal data, and targeted humiliation, often aiming to damage individuals’ reputations, privacy, and psychological well-being. Studies reporting [31,39,41]
***Economic Abuse***	Exploitation of financial systems or online platforms to restrict access to resources, cause economic harm, or exert control. Studies reporting [39–41]

### Type of technology facilitated gender-based violence perpetrated

Below is taxonomy of the principal forms of gender-based violence (GBV) that emerged from the included studies.

#### Cyber-harassment (online hate speech).

Five studies reported cyber-harassment as a common form of TFGBV experienced by women and girls in sub-Saharan Africa [34,36,37,39,42]. Cyber-harassment included repeated sexist, threatening, degrading, or abusive communication delivered through social media platforms, messaging applications, and online discussion spaces. A Tanzanian study reported that women commonly experienced threatening and insulting messages through Facebook, WhatsApp, and Twitter platforms [37]. Similarly, research from Zimbabwe identified sexist online abuse and misogynistic attacks on social media as frequent experiences among women internet users [36]. Another Zimbabwean study found that women were repeatedly exposed to degrading comments, threats, and online intimidation through digital platforms [37].

In Malawi, women described receiving offensive and abusive messages through online communication platforms, particularly social media applications [39]. A study conducted among students in Ghana reported that 73.2% of participants had received offensive text messages, while 31.1% had received abusive emails, indicating that text-based harassment was more prevalent than email-based harassment [42].Overall, cyber-harassment manifested through hate speech, repeated abusive communication, misogynistic comments, threats, and intimidation targeting women and girls online.

#### Cyber-bullying.

Three studies reported cyber-bullying as a manifestation of TFGBV in sub-Saharan Africa [29,31,42]. Cyber-bullying involved repeated hostile online behavior intended to ridicule, shame, threaten, or socially isolate victims. A study among Kenyan university students identified Facebook-based bullying as a common form of online aggression targeting students [31]. Another Kenyan study found that some victims of cyber-victimization later engaged in retaliatory cyber-bullying against others, including gendered online harassment and humiliation [29]. The study also noted that cyber-bullying frequently involved rumor spreading, public ridicule, and online shaming.

Research conducted in Ghana indicated that offensive text messages and abusive digital communication were common among students and often contributed to online bullying experiences [42]. Across the studies, cyber-bullying manifested through online ridicule, rumor dissemination, public humiliation, repeated harassment, and peer-based digital aggression.

#### Cyber-stalking and digital monitoring.

Cyber stalking and digital monitoring were addressed in eleven studies across Sub-Saharan Africa [27–29,31,32,34,37,39,41,44,45]. These behaviors are consistently framed as significant and escalating forms of technology-facilitated gender-based violence (TFGBV). Several studies highlighted university students as disproportionately affected, with reported victimization rates ranging from 49.7% to 75.8%, substantially higher than the 23% reported among secondary school students [27,29,44].The forms of cyber stalking ranged from repeated messaging and digital surveillance to unauthorized access of private accounts and forced password disclosure [32,34]. The role of cyber stalking in intimate partner violence (IPV) was emphasized in multiple studies. Behaviors such as monitoring online activity, GPS tracking, coercing access to digital accounts, and incessant messaging were noted as tools of control and harassment. For instance, among university students, “coercive-control” cyber dating abuse especially via platforms like WhatsApp was identified as the most pervasive form of [28,41]. A study from the University of Dar es Salaam underlined that these practices are not isolated, but rooted in gendered power dynamics and structural inequalities, reinforcing offline patterns of control and abuse [37].

#### Non-consensual intimate-image distribution (NCII); “Revenge porn”.

Seven studies examined non-consensual intimate-image distribution (NCII), commonly referred to as “revenge porn,” as a major manifestation of TFGBV in sub-Saharan Africa [30,32–34,36,39,40]. The studies described NCII as the sharing or threatening to share sexually explicit images without consent to humiliate, blackmail, intimidate, or control victims. In Uganda, survivors and legal stakeholders identified revenge porn as a widespread form of abuse linked to coercion, social humiliation, and intimidation [33]. Another Ugandan study reported that perpetrators frequently used intimate images to threaten and silence women [34]. In Malawi, women described experiences of blackmail involving nude images and threats of public exposure through digital platforms [39]. Research from Zimbabwe found that women experienced the circulation of nude or semi-nude images on social media platforms without consent, often resulting in reputational harm and social stigma [36]. Similarly, a Nigerian study reported that perpetrators distributed intimate images to exploit, shame, or extort women online [40]. Studies from Kenya highlighted how consensually shared intimate images later became tools for abuse within romantic relationships [30,32]. One Kenyan study found that images initially exchanged as expressions of intimacy were later used to manipulate, threaten, or publicly shame women after relationship disputes or separation [32]. Across the included studies, NCII manifested through image-based abuse, online humiliation, blackmail, coercion, reputational attacks, and digital intimidation.

#### Sextortion.

Four studies identified sextortion as an important manifestation of TFGBV in sub-Saharan Africa [37,39–41]. Sextortion involved threats to release intimate images, videos, or private digital conversations unless victims complied with demands for money, sexual favors, or other forms of exploitation. A study conducted in Malawi reported high levels of online exploitation involving threats linked to intimate digital content [39]. In Tanzania, research conducted within higher education institutions found that sextortion was prevalent among female students, with some perpetrators reportedly being academic staff members who used digital communication to coerce students into sexual relationships [37]. Research from Nigeria documented cases in which perpetrators used sexually explicit content to blackmail women for financial gain or sexual compliance [40]. Another study from East Africa described sextortion as a form of coercive digital abuse that could escalate into offline physical and psychological violence [41]. Across the included studies, sextortion manifested through digital blackmail, coercion, exploitation, threats of image release, and abuse of institutional or relational power.

#### Cyber-dating abuse (digital intimate-partner violence).

Five studies reported cyber-dating abuse, also referred to as digital intimate partner violence, as a common manifestation of TFGBV in sub-Saharan Africa [27,28,32,39,40]. The studies described cyber-dating abuse as abusive, controlling, or exploitative behaviors occurring within intimate or romantic relationships through digital technologies and online platforms. Research conducted among university students in Kenya reported a high prevalence of cyber-dating abuse. One study found that 87.3% of students involved in online dating had experienced at least one form of digital abuse [28]. The manifestations of abuse included excessive monitoring of partners’ online activities, coercive messaging, threats, harassment, emotional manipulation, and controlling behaviors through social media and messaging platforms [27,32]. Studies also identified online dating applications and social networking platforms as important pathways facilitating abuse. In Malawi, dating applications were specifically associated with incidents of digital intimate partner violence [39]. Additionally, some studies described cyber-dating abuse in the form of online dating scams, deception, and exploitation targeting women through digital relationships [40]. Overall, the findings indicate that cyber-dating abuse manifests through coercive control, emotional abuse, surveillance, harassment, and exploitation within digitally mediated intimate relationships.

#### Defamation, doxxing and public shaming.

Multiple studies identified defamation, doxxing, and public shaming as interconnected manifestations of TFGBV in sub-Saharan Africa [31,39,41]. These forms of abuse involved the dissemination of false information, unauthorized disclosure of personal information, and deliberate public humiliation intended to damage victims’ reputations, privacy, and psychological well-being Studies from Malawi reported that online defamation was among the most frequently experienced forms of cyber violence against women [39]. Manifestations included spreading false accusations, reputational attacks, and circulation of harmful or misleading information through social media and online communication platforms. Research conducted in Kenya found that women with larger social media networks were more likely to experience public shaming and reputational attacks on platforms such as Facebook [31]. The studies further indicated that doxxing involved exposing personal details, photographs, or identifying information without consent, thereby increasing victims’ vulnerability to harassment and intimidation [41].

### Effect of technology facilitated gender-based violence on victims

#### Psychological impacts of technology-facilitated gender-based violence.

Seven studies [27,32,35,38,39,41,42] reported psychological consequences of technology-facilitated gender-based violence (TFGBV). A study from Kenya and Nigeria found that the majority of respondents experienced depression, anxiety, and fear as a result of victimization [32,38]. Particularly severe are the psychosocial impacts of “revenge porn,” with victims in Zimbabwe and Kenya reporting anxiety, depression, and even suicidal thoughts after discovering that their naked images were viewed by parents, friends, and others [27,35]. Another study highlighted that TFGBV, such as cyber bullying, often leads to psychological symptoms including anxiety and depression [42]. In Malawi, research showed that 49.3% of victims lived in fear, 3% suffered from depression, 1.5% had suicidal thoughts, and 9% experienced anxiety as a result of cyber bullying [39].Additionally, a multi-country study covering sub-Saharan Africa reported that 56% of survivors of TFGBV frequently suffered psychological harm, including anxiety, depression, low self-esteem, fear, and suicidal thoughts [41].

#### Effect on Academic and educational performance.

Evidence from seven studies conducted on students in different countries of sub-Saharan Africa [27,31,32,37,38,42,45] indicated that technology-facilitated gender-based violence extends to the learning process and academic performance. Technology-assisted gender-based violence impact is not limited to psychological consequences; it also extends to the learning process and academic performance. This occurs through the combined psychological impacts, which worsen students’ academic performances, leading to issues like skipping classes, attention deficits, inability to handle assignments timely, and lower grade [32,38,45]. The negative effect of technology-assisted gender-based violence on academic performance is mediated by the depression and fear it imposes, and when it involves different types of TFGBV like revenge porn, it even leads to shame and embarrassment of facing classmates [27]. Moreover, even school deans have reported that they spend their time resolving cases related to online GBV in Kenya [31].

#### Social and reputational damage.

Six studies [30,33,36,37,39] reported the social impact and reputational damage associated with technology-facilitated gender-based violence (TFGBV). Several studies reported effects such as social withdrawal [30,33,37,39]. In Malawi [39]found that 68.7% of victims withdrew from online activities due to public defamation and shaming, 17.2% reported loss of reputation, 9% disengaged from social activities, 3% became isolated from colleagues and family, and 1.5% relocated from their communities. Similarly, findings from Kenya indicate that even women and girls who voluntarily shared nude images within romantic relationships later reported feelings of insecurity [30]. Reputational damage was linked to the erosion of personal dignity among victims [37]. Victims are often engaged in social withdrawal as a self-preservation measure, potentially reducing women’s and girls’ contributions to development via digital platforms [33],Perpetrators were sometimes perceived as “champions,” while victims were labelled with stigmatizing terms contributing to victims’ reluctance to seek help and resulting in silent suffering [33,36] specifically finding from sub-Saharan African countries indicates that only 5% seek counseling while 32% responded by blocking offender [40]*.*

#### Help-seeking barriers and long-term consequence.

Finally, four studies [27,37,39,40] identified multiple barriers that hindered victims from seeking help. Personal barriers included shame, stigma, lack of awareness, and digital disengagement, which sometimes led victims to interpret online interactions as trust rather than risk [27,37,39]. Social and cultural barriers such as patriarchal norms and lack of community support, alongside institutional barriers like inadequate platform interventions and victim-blaming attitudes, further reduced help-seeking behavior. As a result, only 5% of victims sought counseling, while 32% choose for blocking offenders as their primary coping strategy [37,39,40].

#### Economic impact.

Three studies reported economic abuse as a consequence and manifestation of TFGBV in sub-Saharan Africa [39–41]. The studies described economic abuse as the use of digital technologies to cause financial harm, restrict economic opportunities, or exploit victims financially. Studies from Uganda and Rwanda reported that approximately 32% of participants experienced economic impacts related to TFGBV, including restrictions on internet use for entrepreneurship, business activities, and financial advancement. In Rwanda, the non-consensual leaking of intimate images, particularly when victims’ identities were revealed, resulted in job loss or voluntary resignation due to stigma and psychological distress [41].

Research from Nigeria described how perpetrators used impersonation tactics, online scams, and financial extortion to target women entrepreneurs and models through social media platforms. [40].In Malawi, women reported blackmail involving intimate images, leading to significant financial consequences; 76.1% reported income loss, while 12% reported difficulty obtaining new employment following victimization [39]. These findings demonstrate that economic abuse manifests through financial extortion, employment disruption, reputational damage affecting livelihoods, and restriction of women’s economic participation through digital abuse.

**Fig 2:**
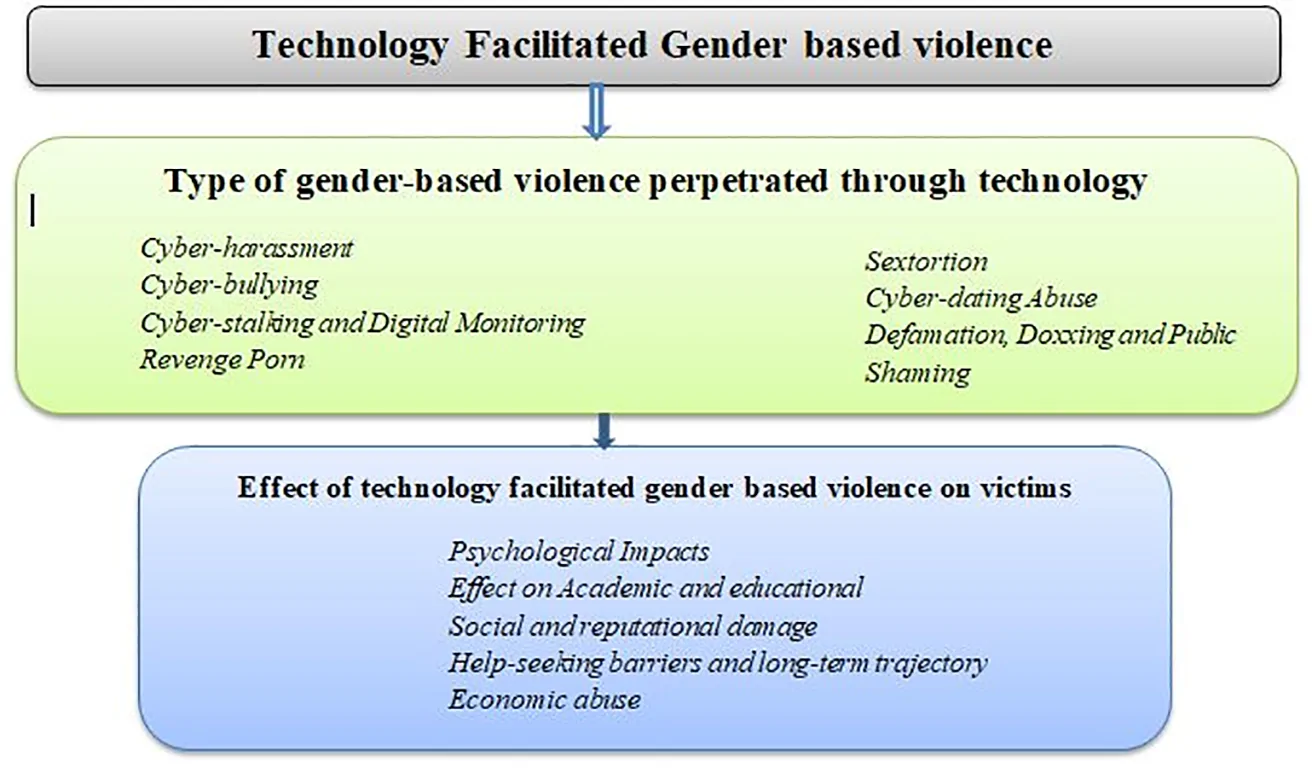
Conceptual Framework of the review of technology facilitated gender based violence in sub-Saharan Africa.

Generally, the types of gender-based violence perpetrated against women and girls, along with their effects in sub-Saharan Africa, are illustrated in Fig 2.

## Discussion

### Summary of major findings

This scoping review aimed to explore how technology facilitates gender-based violence and the consequences of technology-facilitated gender-based violence (TFGBV) in sub-Saharan Africa. As a relatively emerging issue, the first studies on TFGBV in the region were published in 2019. A range of TFGBV types were identified, including cyber-harassment, cyberstalking, cyber bullying, sextortion, cyber dating abuse, defamation, doxxing and public shaming, and economic abuse. The consequences for victims include psychological harm, educational disruption, reputational and social damage, and long-term barriers to seeking help.

Compared with evidence from high-income settings, TFGBV in sub-Saharan Africa appears to be more strongly shaped by structural inequalities, patriarchal gender norms, weak legal protections, limited digital literacy, and barriers to formal reporting systems. While studies from Europe and North America often emphasize platform governance, privacy concerns, and individual-level risk factors [8,9,15] from sub-Saharan Africa more frequently highlighted coercive control, social stigma, institutional power imbalances, economic vulnerability, and normalization of digital surveillance within intimate relationships [35,36,41,46,47]. These differences may reflect variations in legal frameworks, gender relations, access to support systems, digital safety awareness, and broader sociocultural contexts across regions.

### Type of technology facilitated gender-based violence perpetrated

Cyber-harassment, cyberbullying, and cyberstalking emerged as the most frequently reported forms of TFGBV. These included repeated online abuse, hate speech, intimidation, surveillance, threats, and unauthorized sharing of personal information [34,36,37,46,48–50].Similar to international evidence, anonymity and accessibility of digital platforms enabled perpetrators to repeatedly target victims with limited accountability [33,35–37]. Perpetrators also used digital tools such as GPS tracking, spyware, shared passwords, and messaging applications to monitor and control victims [41,51,52]. In sub-Saharan Africa, underreporting appeared to be reinforced by patriarchal norms, stigma, limited awareness, and weak legal protections [11,19,47]. The review further identified growing concerns related to non-consensual image sharing, sextortion, and cyber-dating abuse, particularly within contexts characterized by poverty, corruption, and rapid digitalization without adequate safeguards [37,39–41,52–56]. Although these forms of abuse are increasingly recognized globally, evidence from sub-Saharan Africa remains limited.

The review also highlighted the public-facing dimensions of TFGBV, including defamation, doxxing, body-shaming, and slut-shaming [57–59]. These forms of abuse disproportionately targeted women in public roles and women perceived to challenge social norms [60]. Technology amplified these harms through rapid dissemination, permanence of content, and mass participation in online harassment. Together, these findings demonstrate that TFGBV is not only interpersonal violence facilitated by technology, but also a broader manifestation of gendered power inequalities reproduced in digital spaces.

### What are the effects of TFGBV on victims?

TFGBV had substantial psychological, educational, social, and economic consequences. Victims frequently experienced anxiety, depression, emotional distress, social withdrawal, and reduced self-esteem [61–63]. Severe psychological outcomes, including suicidal ideation and sleep disorders, were also reported [64]. Educational impacts included absenteeism, disengagement from learning, reduced academic performance, and disruptions to educational trajectories [10,13,65]. Survivors of image-based abuse and online shaming often experienced stigma, reputational damage, and exclusion from social and professional spaces [9,10,64]. Some victims reported withdrawing from employment, education, or online participation because of ongoing harassment and fear of further exposure [9,10,61,64].

The review further showed that TFGBV creates substantial barriers to help-seeking. Fear of stigma and social repercussions, limited awareness of reporting mechanisms, distrust in authorities, patriarchal norms, family pressure, and digital exclusion frequently delayed or prevented access to support services [11,20,53,66].These barriers contributed to prolonged psychological distress and reduced opportunities for recovery. Economic abuse also illustrated how digital technologies can be weaponized to undermine financial autonomy through surveillance, restriction, and coercive control of digital financial systems and online workspaces [19,33]. Weak digital regulation and unequal digital literacy further increased vulnerability and limited accountability [15,16,67,68].

## Limitations

The findings of this review have important implications for policy and programming in sub-Saharan Africa. TFGBV should be explicitly integrated into national gender-based violence frameworks, cybercrime legislation, and digital governance policies. Strengthening survivor-centered reporting systems, legal protections, and platform accountability mechanisms is essential. The findings also support the integration of digital literacy and online safety education into schools, universities, and community-based programs, particularly for adolescents and young women. In addition, mental health care, legal support, and digital safety services should be incorporated into existing gender-based violence response systems.

### Evidence gaps for future research

As a scoping review, this study also identified several important gaps in the evidence base. Most included studies originated from a small number of countries, particularly Kenya, Uganda, Malawi, Tanzania, and Nigeria, leaving substantial geographic gaps across the region. Evidence was also concentrated among university students and younger populations, with limited attention to rural communities, older adults, adolescents outside educational settings, and individuals with limited internet access. Variations in terminology, measurement tools, and reporting approaches further limited comparability across studies. Future research should prioritize standardized definitions, validated measurement tools, and more consistent reporting approaches to strengthen evidence synthesis.

Additional evidence is also needed regarding platform-specific risks and mechanisms. Although Facebook and WhatsApp were commonly discussed [28,31,32,36,39,40] limited evidence examined platforms such as Instagram, TikTok, Snapchat, Telegram, Signal, livestreaming services, and mobile-money applications. Future studies should examine how features such as anonymity, encryption, algorithmic amplification, and disappearing messages shape TFGBV risks. Importantly, evidence on escalation pathways from online abuse to offline violence remains extremely limited. Only one included study explicitly reported progression from online gender-based violence to physical violence [69]. Longitudinal and mixed-methods research is therefore needed to better understand escalation pathways, long-term consequences, and effective interventions.

## Conclusion

TFGBV in sub-Saharan Africa represents a multidimensional form of violence that extends beyond online spaces to affect psychological well-being, education, social participation, economic security, and access to support services. Although the identified patterns share similarities with global evidence, TFGBV in the region is strongly shaped by structural inequalities, patriarchal norms, limited digital protections, and barriers to help-seeking. Addressing TFGBV therefore requires coordinated and context-sensitive responses involving legal reform, survivor-centered services, digital literacy initiatives, stronger platform accountability, and sustained investment in research and policy development.

## Supporting information

S1 File Items for systematic reviews and meta-analyses extension for scoping reviews (PRISMA-ScR). (DOCX)

S2 File Detailed search strategies for each database. (DOCX)

S3 File Literatures excluded with reasons. (DOCX)
