# Supplementary material for: Technology-facilitated gender-based violence against women and girls in sub-Saharan Africa: A scoping review
Source: PLoS One. 2026 Aug 19;21(8):e0354526. doi: 10.1371/journal.pone.0354526 (PMC13489429; doi:10.1371/journal.pone.0354526)
Supplement: Supplementary file 2 [file pone.0354526.s002.docx]

Data bases and search term used

| Data bases | Search terms | Studies |
| --- | --- | --- |
| PubMed | *("Gender-Based Violence"[Mesh] OR "Sexual Trauma"[Majr] OR bullying OR harassment OR "online abuse") AND ("Smartphone"[Mesh] OR "Mobile Applications"[Mesh] OR "Information Technology"[Majr] OR "Internet Use"[Majr]) AND "Africa South of the Sahara"[Mesh]* | 53 |
| Scopus | TITLE-ABS-KEY ( ( "technology-facilitated" ) ) OR ( "Information Technology" ) AND ( ( "Gender-Based Violence" ) OR ( "Sexual Trauma" ) ) AND ( LIMIT-TO ( SUBJAREA , "MEDI" ) OR LIMIT-TO ( SUBJAREA , "NURS" ) OR LIMIT-TO ( SUBJAREA , "ENGI" ) OR LIMIT-TO ( SUBJAREA , "PSYC" ) OR LIMIT-TO ( SUBJAREA , "SOCI" ) OR LIMIT-TO ( SUBJAREA , "COMP" ) ) AND ( LIMIT-TO ( EXACTKEYWORD , "Gender-based Violence" ) OR LIMIT-TO ( EXACTKEYWORD , "Intimate Partner Violence" ) OR LIMIT-TO ( EXACTKEYWORD , "Gender" ) OR LIMIT-TO ( EXACTKEYWORD , "Psychology" ) OR LIMIT-TO ( EXACTKEYWORD , "Female" ) ) AND ( LIMIT-TO ( DOCTYPE , "ar" ) ) | 11 |
| Science Direct | (“Cyber-based gender-based violence” OR “online gender-based violence” OR “internet-based harassment” OR” social media based sexual violence” OR “digital harassment” OR “cyber violence” OR “Cyber bullying of women” OR “online misogyny“ OR “image-based abuse” OR “Non-consensual intimate image sharing” OR “ICT-based harassment” OR “women deep faking”) | 201 |
| EBESCO | ("Gender-Based Violence"[Mesh] OR "Sexual Trauma"[Majr] OR bullying OR harassment OR "online abuse") AND ("Smartphone"[Mesh] OR "Mobile Applications"[Mesh] OR "Information Technology"[Majr] OR "Internet Use"[Majr]) AND "Africa South of the Sahara"[Mesh] | 13 |
| Google scholar | “technology-facilitated gender-based violence” OR  “technology-assisted violence against women” | 56 |
| AJOL | “technology-facilitated gender-based violence” OR  “technology-assisted violence against women” | 10 |
