# Supplementary material for: Technology-facilitated gender-based violence against women and girls in sub-Saharan Africa: A scoping review
Source: PLoS One. 2026 Aug 19;21(8):e0354526. doi: 10.1371/journal.pone.0354526 (PMC13489429; doi:10.1371/journal.pone.0354526)
Supplement: Supplementary file 3 [file pone.0354526.s003.docx]

Excluded studies with reason on full text review

| No |  | Country | Title | Reason of exclusion |
| --- | --- | --- | --- | --- |
| 1 | (De Filippo et al., 2023) | South Africa | Effects of digital chatbot on gender attitudes and exposure to intimate partner violence among young women in South Africa | **It focuses on GBV prevention** |
| 2 | (Martin, 2021) | Nigeria | The Mass Media and Gender-Based Violence in Imo State, Nigeria: A Critical Analysis | **Focus on GBV prevention by media/** |
| 3 | (Agidew, 2023) | Ethiopia* | Online gender-based violence under the law of Ethiopia: a comparative approach | policy research |
| 4 | (Mulisa & Getahun, 2018) | Ethiopia | Perceived Benefits and Risks of social media: Ethiopian Secondary School Students’ Perspectives | **Not addressed TFGBV** |
| 5 | (Lachapelle et al., 2022) | Canada | A Mixed Methods Approach Exploring Risk Factors Associated with Cyber Dating Victimization and Resilience in Adolescents and Emerging Adults | **Different setting** |
| 6 | (Lachapelle et al., 2022) | Indian | The Role of Law in Combatting Gender-Based Violence on social media and Online Platforms | **Different setting** |
| 7 | (UN Women, 2020) | Global | Background Paper-Safe Digital Spaces: Protection of Women and Girls from Technological Violence | Policy review |
| 8 | (Soehnchen et al., 2024) | Kenya | A Digital Sexual Health Education Web Application for Resource-Poor Regions in Kenya: Implementation-Oriented Case Study Using the Intercultural Research Model | **It focuses on GBV prevention/**  Digital sexual health |
| 9 | (Amaliya et al., 2024) | Indonesia | Contemporary Perceptions of Gender Due To Online Gender-Based Violence (Ogbv) in the Digital Society | **Different setting** |
| 10 | (Peega et al., 2025) | South Africa | Students’ Perceptions of Factors Contributing to Violence Against Women: A Case of One University in the Gauteng Province of South Africa | **Don’t addressed TFGBV** |
| 11 | (Leitão, 2021) | UK | Technology-Facilitated Intimate Partner Abuse: a qualitative analysis of data from online domestic abuse forums | **Setting** |
| 12 | (Waterman et al., 2021) | Zimbabwe | Zimbabwean Stakeholders’ Perspectives on Causes of and Solutions to Gender-Based Violence in Their Community: Findings From a Focus Group | **Don’t’ addressed TFGBV** |
| 13 | (Okonofua et al., 2024) | Nigeria | Applying Mobile Technology to Address Gender-Based Violence in Rural Nigeria: Experiences and Perceptions of Users and Stakeholders | **Prevention of GBV** |
| 14 | (Silva et al., 2023) | Nigeria | Gender-Based Violence Narratives in Internet-Based Conversations in Nigeria: Social Listening Study | **different population** |
| 15 | (Kwanya et al., 2022) | Kenya | Cyber-bullying research in Kenya: a meta-analysis | **Not addressed TFGBV** |

*from gray literature
